# 3D-printed tricalcium phosphate scaffolds with Quercetin-zinc chelation for osteogenic, antibacterial and anti-osteosarcoma activity

**DOI:** 10.1093/rb/rbag139

**Published:** 2026-10-16

**Authors:** Susmita Bose, Connor Toulou, Aditi Dahiya

**Affiliations:** W. M. Keck Biomedical Materials Research Laboratory, School of Mechanical and Materials Engineering, Washington State University, Pullman, WA 99164, USA; W. M. Keck Biomedical Materials Research Laboratory, School of Mechanical and Materials Engineering, Washington State University, Pullman, WA 99164, USA; W. M. Keck Biomedical Materials Research Laboratory, School of Mechanical and Materials Engineering, Washington State University, Pullman, WA 99164, USA; Department of Chemistry, Washington State University, Pullman, WA 99164, USA

**Keywords:** 3D-printing, quercetin, chelation, calcium phosphate, infection control

## Abstract

Craniomaxillofacial bone defects and implant-related infections require materials that support regeneration while providing localized therapeutic action. Quercetin offers osteogenic, antibacterial, and anticancer activity, but poor stability and bioavailability limit direct use. To address this, a Quercetin–zinc (Q–Zn^2+^) complex is loaded onto 3D-printed (3DP) tricalcium phosphate (TCP) scaffolds, where zinc coordination stabilizes Quercetin through metal-ligand interactions and enables controlled release. The release behavior of Q–Zn^2+^ from 3DP TCP and its antibacterial effects remain unexplored. Characterization of the complex shows that Zn^2+^ chelates with Quercetin’s hydroxyl and carbonyl groups. Release studies at pH 5.0 and pH 7.4 show sustained delivery of Quercetin over 24 days, reaching 16% release at pH 5.0 and 5% at pH 7.4 for complexed Quercetin. *In vitro* evaluation shows that Q–Zn^2+^-loaded scaffolds increase osteoblast viability by 42%, reduce osteosarcoma viability by ∼55%, and achieve 93% inhibition of *Staphylococcus aureus* compared to TCP control. Zn^2+^ chelation stabilizes Quercetin release while improving osteoblast viability and suppressing bacterial and osteosarcoma growth from 3DP TCP scaffolds. This approach offers a route for controlled therapeutic delivery of Quercetin in calcium phosphate bone graft materials.

## Graphical abstract

**Graphical Abstract:**
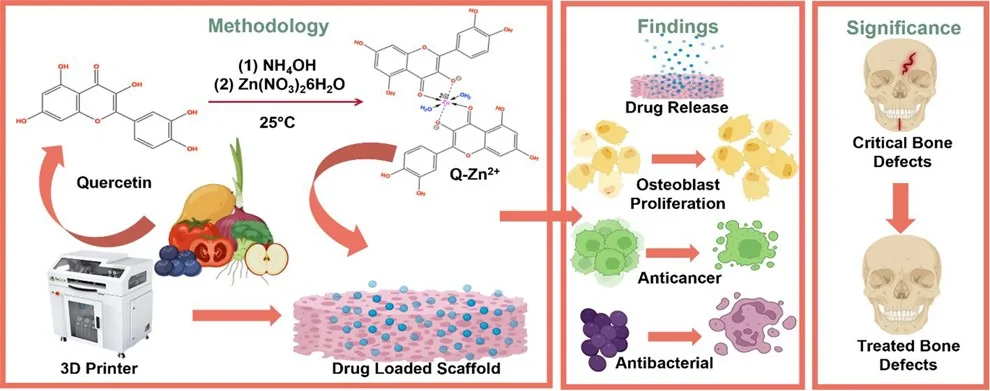

## Introduction

Craniomaxillofacial (CMF) defects resulting from trauma, surgery, or infections present clinical challenges, with over 7,000,000 new cases reported annually [1]. Recovery from CMF repair is often prolonged, taking approximately 60 days for complete bone healing, and can place a substantial psychological and economic burden on patients [2]. Traditional treatment strategies, such as autografts and allografts, aim to restore the musculoskeletal framework but are limited by donor site morbidity (<10.8%), infection risks (<3%), and high failure rates (9.4% for autografts and 26.5% for allografts) [3–5]. To overcome these limitations, current research is increasingly focused on enhancing the body’s regenerative capacity through localized delivery of bioactive molecules that can accelerate bone healing.

Natural medicinal compounds have recently garnered interest due to their antibacterial, osteogenic, and anti-inflammatory properties in bone repair [6]. Among them, Quercetin is promising due to its antioxidant activity and ability to stimulate osteoblast (OB) differentiation and inhibit osteoclastogenesis [7–9]. Oral delivery of Quercetin has been studied in clinical trials; however, local delivery via grafting requires further preclinical study [10]. The therapeutic application of Quercetin is limited by its low water solubility, rapid degradation, and high burst release when incorporated into scaffolds. To mitigate the burst release of Quercetin, several strategies have been explored. For instance, our group previously reported that incorporating KCl into tricalcium phosphate (TCP) scaffolds via extrusion could partially modulate the initial burst release profile of Quercetin [11]. Other researchers have attempted nanoparticle encapsulation approaches to slow down the release, while additional methods such as polymeric coatings, tuning scaffold porosity/surface chemistry, amino acid conjugates, and nanoparticle encapsulation have also been investigated [12–14]. Despite these efforts, complete control over Quercetin release remains challenging. We therefore propose that metal–Quercetin complexation could offer a more robust alternative, as chelation may enhance stability, suppress premature degradation, and enable a more sustained release [15]. Previous work showed that chelating curcumin with vanadium suppressed hydrolytic cleavage under physiological conditions, improving both bioavailability and sustained release [16]. Additionally, Quercetin-loaded zeolite imidazolate framework-8 nanoparticles utilized a zinc shell for immunoregulation of tissue-engineered bone [17]. Similarly, zinc, a trace element essential for bone metabolism, stabilizes Quercetin through chelation but also enhances osteogenesis by stimulating alkaline phosphatase activity and mineralization [18–20]. A clinical trial conducted in 2015 demonstrated that zinc supplementation increased procollagen type 1 amino-terminal propeptide levels in adolescent women, supporting its role in bone formation [21]. Given that zinc is required only in trace amounts, with recommended daily intakes of 8 mg/day for women and 11 mg/day for men (US Institute of Medicine), controlled release from biomaterials becomes essential to avoid toxicity while maintaining therapeutic effects [22]. This dual role makes Q–Zn^2+^ complexation an attractive, but not yet fully resolved, strategy for local therapeutic delivery in bone repair. Prior studies have already established that Q–Zn^2+^ complexes possess biological activity in several non-scaffold systems. For example, Q–Zn^2+^ complexes have shown enhanced anticancer activity relative to free Quercetin in HepG2, HCT116, and MG63 cells, with increased apoptosis and improved intracellular uptake of both Quercetin and zinc [23–25]. Other studies have examined Q–Zn^2+^ or zinc-Quercetin systems in antioxidant, antidiabetic, and osteogenic contexts, supporting the broader therapeutic relevance of this chelation approach [25, 26]. However, the stability of Quercetin–metal complexes is medium-dependent, where aqueous studies show that Quercetin can chelate transition metals at physiological pH, but complexation may also be accompanied by metal-mediated oxidation or degradation depending on the ion, pH, and coordination environment [27]. Therefore, Q–Zn^2+^ complexation should not be treated simply as a universally stabilizing modification, but rather as a formulation strategy whose behavior must be evaluated in the intended delivery matrix. The incorporation of a Q–Zn^2+^ complex into 3DP TCP scaffolds remains insufficiently characterized. In particular, the effects of the TCP scaffold environment on Q–Zn^2+^ release kinetics, OB compatibility, osteosarcoma (OS) response, and antibacterial activity have not been systematically evaluated. However, Q–Zn^2+^ release behavior from 3DP TCP scaffolds and its antibacterial effects have not been explored.

TCP is widely used as a synthetic bone substitute due to its biocompatibility, osteoconductivity, and ability to resorb and be replaced by natural bone [28, 29]. While TCP’s surface area and porosity facilitate cell attachment and tissue integration, its therapeutic potential can be further enhanced by loading it with bioactive molecules [30–32]. Here, we investigate the controlled release of a Q–Zn^2+^ complex from 3DP TCP scaffolds, aiming to couple the osteogenic benefits of Quercetin with the sustained delivery afforded by zinc complexation. This study answers several scientific questions: (i) Can chelation of Quercetin with Zn^2+^ enhance its physicochemical stability and enable pH-responsive, sustained release from 3DP-TCP scaffolds? (ii) How does metal-ligand complexation influence the interaction of the scaffold with OB and OS proliferation? (iii) Does Zn^2+^ chelation impart synergistic antibacterial and osteogenic effects, improving the scaffold’s multifunctionality for bone tissue regeneration? This study hypothesizes that the complexation of Quercetin with Zn^2+^ will enhance cell proliferation and subsequent ALP production in OBs, while suppressing OS and *S. aureus*. The Q–Zn^2+^ chelation is hypothesized to mitigate the initial burst release from a scaffold substrate.

## Materials and methods

### Fabrication of 3DP TCP scaffolds

β -TCP was synthesized using solid-state synthesis. Calcium phosphate dibasic and calcium carbonate were mixed in a 2:1 ratio and then ball-milled at 70 rpm for 2 h in a 1:2 ratio of powder to milling media. The powder was then calcined at 1050°C for 24 h. After calcination, the powder was mixed with ethanol in a 1:1.5 ratio and ball-milled at 70 rpm for 6 h. The resulting mixture was washed 6 times to remove any precipitated salt and then dried at 70°C for 24 h.

The synthesized powder was utilized to fabricate 3DP scaffolds, which were designed using computer-aided design (CAD) software. The 12.5-mm diameter and 2-mm height scaffolds were printed using a binder jet printer (ProMetal, ExOne LLC, PA, USA). After printing, the scaffolds were cured by heating at 195°C for 2 h to solidify the binder, and the scaffolds were depowdered by blowing air. The scaffolds were then sintered in a conventional muffle furnace at 1250°C for 2 h to complete the fabrication process [19]. The scaffolds were autoclaved at 120°C for 1 h then aseptically transferred to the sterile fume hood. For cell culture, Quercetin and Q–Zn^2+^ were loaded onto the scaffolds by drop-casting from ethanol-based solutions that were passed through a 0.22-µm filter to ensure sterility. The concentration of each drug solution depended on the target loading amount for the corresponding OB, OS, or antibacterial experiment and is specified in the relevant section. To improve solution uniformity before loading, drug solutions were vortex-mixed to disperse the drug and then mixed overnight using a magnetic stir bar prior to drop-casting [33].

### Quercetin–Zn^2+^ complexation

A solution containing 1 mmol of Quercetin (Sigma Aldrich, ≥ 95% purity by HPLC) was dissolved in 30 mL of methanol, followed by adjusting the pH to 8.0 using 1 M ammonium hydroxide. Then, 0.7 mmol of zinc nitrate hexahydrate (Zn (NO_3_)_2_·6H_2_O) was added to the same solution. The solution was mixed continuously for 4 h at 20°C. The product was formed and filtered to obtain the solid complex (34% yield). The solid was washed with methanol to purify the product. Complex purity was confirmed by ^1^H NMR spectra, Fourier transform infrared spectroscopy (FTIR), and UV-Vis matched literature [20].

All calculations were performed using the GFN2-xTB method as implemented in the xtb software package to find the most stable conformation of the complex [34]. The model system consisted of one Zn^2+^ ion, two deprotonated Quercetin ligands, and two H_2_O molecules, forming an overall neutral complex. Geometry optimization was carried out in the implicit aqueous phase using the Generalized Born Surface Area (GBSA) solvation model with standard water parameters [35]. The optimization was performed without symmetry constraints until the maximum gradient was below 10^−5^ E_h_ a_0_^−1^. Vibrational frequency analysis was subsequently performed at the same level of theory to confirm the absence of imaginary frequencies, ensuring that the optimized geometry corresponds to a true local minimum on the potential energy surface.

### *In vitro* Quercetin release

Quercetin (60 μg) and Q–Zn^2+^ complex (60 μg) release profiles from 3DP TCP scaffolds were studied at pH 5.0 (acetate buffer) and pH 7.4 (phosphate buffer) to simulate acidic conditions post-surgery and physiological conditions, respectively. Samples loaded with treatment were immersed in 4 mL of buffer solutions and then placed on a shaker at 150 rpm at 37°C to simulate human body conditions. Periodic replacement of the buffer solutions was performed at predetermined intervals ranging from 30 minutes to 24 days. At 0.5, 1, 2, 4, 8, 12, and 24 h, followed by days 2, 3, 5, 7, 14, 21, and 24, Quercetin release was quantified using a Biotek Synergy 2 SLFPTAD microplate reader at 370 nm [36]. The data obtained were plotted as cumulative drug release over time, calculated according to the calibration curve, and the release profiles were mathematically fitted to the Weibull and saturated exponential models using Excel.

### *In vitro* OB cell culture

Human fetal osteoblast (hFOB) cell lines (ATCC, USA), passage 8, were cultured in T-75 flasks using Dulbecco’s Modified Eagle’s Medium (DMEM) supplemented with 10% fetal bovine serum (ATCC, USA) and 1% penicillin/streptomycin (ATCC, USA). A 5-day OB effective concentration study was conducted to determine the optimal loading for each drug. TCP scaffolds were used as the control group for experiments, while treatments included 8 μg of Quercetin and 10 μg of Q–Zn^2+^ per sample [11]. The amount of the complex was determined using an effective concentration study (Supplementary Figure S1). For the full study, 25 × 10^3^ cells were seeded onto each scaffold in a 24-well plate and cultured in DMEM for 11 days under 5% CO_2_ at 37°C [37].

Cellular mitochondrial activity was assessed using an MTT (3-(4,5-dimethylthiazol-2-yl)-2,5-diphenyl tetrazolium bromide) assay at each time point (3, 7, and 11 days). Samples were transferred to new wells, and a 5 mg/mL MTT solution in phosphate buffer was added. The samples were then incubated for 4 h. The resulting formazan crystals were dissolved using a solution containing 10% Triton X-100, 0.1 N hydrochloric acid, and isopropanol. The absorbance of each sample, measured in triplicate, was determined at 570 nm using a UV-Vis microplate reader [38].

On day 11, ALP activity was evaluated using the SensoLyte pNPP ALP Assay Kit (AnaSpec, CA, USA). Samples were initially washed with 1× buffer, and cells were lysed using 1% Triton X-100. The suspension was then collected, stored at 4°C for 10 minutes, and centrifuged at 5500 rpm for 10 minutes. The supernatant was transferred into a 96-well plate, and 50 μL of pNPP (para nitrophenyl phosphate) solution was added to each well. Plates were incubated at 37°C for 45 minutes. The absorbance was measured at 405 nm using a multi-well plate reader [39].

For FESEM, samples were fixed using 2% paraformaldehyde and 2% glutaraldehyde, followed by a post-fixation with 2% osmium tetroxide. Serial dehydration using hydroethanolic solutions was performed, followed by overnight drying with hexamethyldisilazane. Samples were then gold-coated, and images were collected using an FEI Quanta 200F (FEI Inc., OR, USA) at the day 11 time point [40].

For live/dead staining, Calcein-AM (Biolegend, USA) (200 μL) and propidium iodide (Fisher Scientific, USA) (200 μL) were used to stain the cells on day 7, and the cells were imaged using a confocal microscope (TCS SP5, Leica, Germany). Confocal images of the live/dead samples were collected in triplicate, and the percent area quantification was performed using ImageJ [11].

### *In vitro* OS cell culture

Substrate compositions remained the same as mentioned in the above cell culture with Quercetin and Q–Zn^2+^ loading of 87.5 μg. Lower loadings were used for hFOB OB studies to assess cytocompatibility and osteogenic response, whereas the higher 87.5 µg loading was selected for OS and antibacterial studies based on prior Quercetin-loaded TCP scaffold work showing anti-OS activity at this dose [11]. Q–Zn^2+^ was therefore tested at the same total loading to enable direct weight-to-weight comparison with Quercetin and evaluate whether zinc chelation enhanced therapeutic activity. A cell density of 2.5 × 10^4^ MG-63 OS cells was loaded onto each sample with Eagle’s Minimum Essential Media and incubated at 37°C with 5% C$O_{2}$ [41]. Cellular evaluations were performed through MTT assay (done at all the time points), FESEM (day 11), and live/dead staining using the previously discussed protocols (see section “*In vitro* OB cell culture”). The JC-1 staining assay was performed to characterize mitochondrial potential and its effect on cell apoptosis on day 11 of the cell culture. Samples were transferred from the well plate and washed three times with a 1× phosphate buffer solution. The samples were then incubated with JC-1 dye at a concentration of 2 µM for 20 minutes and imaged using a confocal microscope [42].

### *In vitro* antibacterial cell culture

The antibacterial effects of Quercetin and the complex were studied against *S. aureus* bacteria (Carolina Biological Supply Company, Burlington, NC). Quercetin is reported to exhibit antibacterial effects when used at a concentration of 87.5 μg [9, 11]. Therefore, we used the same amount of the complex. The scaffolds were autoclaved and then drug-loaded using a drop-casting method. Antibacterial activity was evaluated by the ISO 22196: 2011 standard, with assessments conducted at 36 and 72 h time points. The optical densities of various bacterial dilutions, measured at 625 nm using a BioTek microplate reader, were referenced against the McFarland standard. Bacteria were first diluted to $10^{4}$ (for agar plate analysis) and $10^{2}$ colony-forming units (CFUs) (for FESEM and live/dead staining) per mL in broth media and then seeded onto the scaffolds. At each time point, the scaffolds were scraped of bacteria and diluted to $10^{2}$ for agar plate analysis. The diluted bacteria were then loaded onto agar plates in triplicate and placed in an incubator at 37°C for 12 h before imaging. The CFUs grown during this time were imaged and counted with an optical camera. Antibacterial efficacy was calculated using Equation (1) [43]. The samples for SEM and live/dead staining were processed according to the section “*In vitro* OB cell culture”.


(1) $$\mathrm{Antibacterial}\;\mathrm{efficacy}\;(\%)=\;\frac{N_{\mathrm{control}}-N_{\mathrm{treatment}}}{N_{\mathrm{control}}}\times 100\%$$

### Statistical analysis

All experimental compositions were tested in triplicate, and the data are represented with mean ± standard deviation. For comparisons, the Student’s *t*-test was used with *P-*value of <0.05, 0.01, and 0.001 considered significant and represented by *, **, and ***, respectively.

## Results

### Complexation of Quercetin–Zn^2+^

The synthesis pathway for the Q–Zn^2+^ complex is illustrated in Figure 1A. Quercetin was reacted with Zn(NO_3_)_2_·6H_2_O in the presence of NH_4_OH at room temperature, producing a stable complex through coordination with Zn^2+^. The process is depicted in Figure 1B, showing the stepwise addition of reagents and subsequent drying to yield 34% of the Q–Zn^2+^ complex. The FTIR spectra for both Quercetin and the Q–Zn^2+^ complex are shown in Figure 1C. Quercetin exhibited a broad peak at 3405 cm^−1^, corresponding to –OH stretching, which shifted to 3382 cm^−1^ in the Q–Zn^2+^ complex, indicating an interaction between Quercetin and Zn^2+^ ions. A shift in the carbonyl (C=O) stretching band was observed, moving from 1672 cm^−1^ in Quercetin to a reduced intensity in the Q–Zn^2+^ complex. These shifts indicate the successful coordination of Quercetin’s hydroxyl and carbonyl groups with Zn^2+^, confirming the formation of a complex. The UV-Vis absorption spectra in Figure 1D display distinct differences between Quercetin and the Q–Zn^2+^ complex. Quercetin showed a characteristic absorption peak at 370 nm, associated with its conjugated π-electron system. A shift in the absorption spectrum was observed upon complexation with zinc, indicating potential changes in the electronic environment due to coordination with Zn^2+^. This shift underscores the formation of the Q–Zn^2+^ complex and suggests alterations in the electronic structure of Quercetin upon binding with Zn^2+^. The results of the ^1^H NMR of both Quercetin and Q–Zn^2+^ are shown in Figure 1E. The Quercetin compound displayed peaks at ^1^H NMR (500 MHz, DMSO) δ 12.50 (s, 1H), δ 10.78 (s, 1H), δ 9.60 (s, 1H), δ 9.38 (s, 1H), δ 9.31 (s, 1H), δ 7.58 (s, 1H), δ 7.55 (s, 1H), δ 6.89 (s, 1H), δ 6.41 (s, 1H), δ 6.19 (s, 1H). Peaks for the Q–Zn^2+^ complex were observed to be ^1^H NMR (500 MHz, DMSO) δ 10.95 (s, 2H), δ 9.76 (s, 2H), δ 9.54 (s, 2H), δ 9.48 (s, 2H), δ 7.84 (s, 2H), δ 7.71 (s, 2H), δ 7.06 (s, 2H), δ 6.57 (s, 2H), δ 6.35 (s, 2H). The disappearance of peak δ 12.50 ppm in the complex indicates a change in electron density around the hydroxyl group, suggesting coordinated bonding with the Zn^2+^.

**Figure 1:**
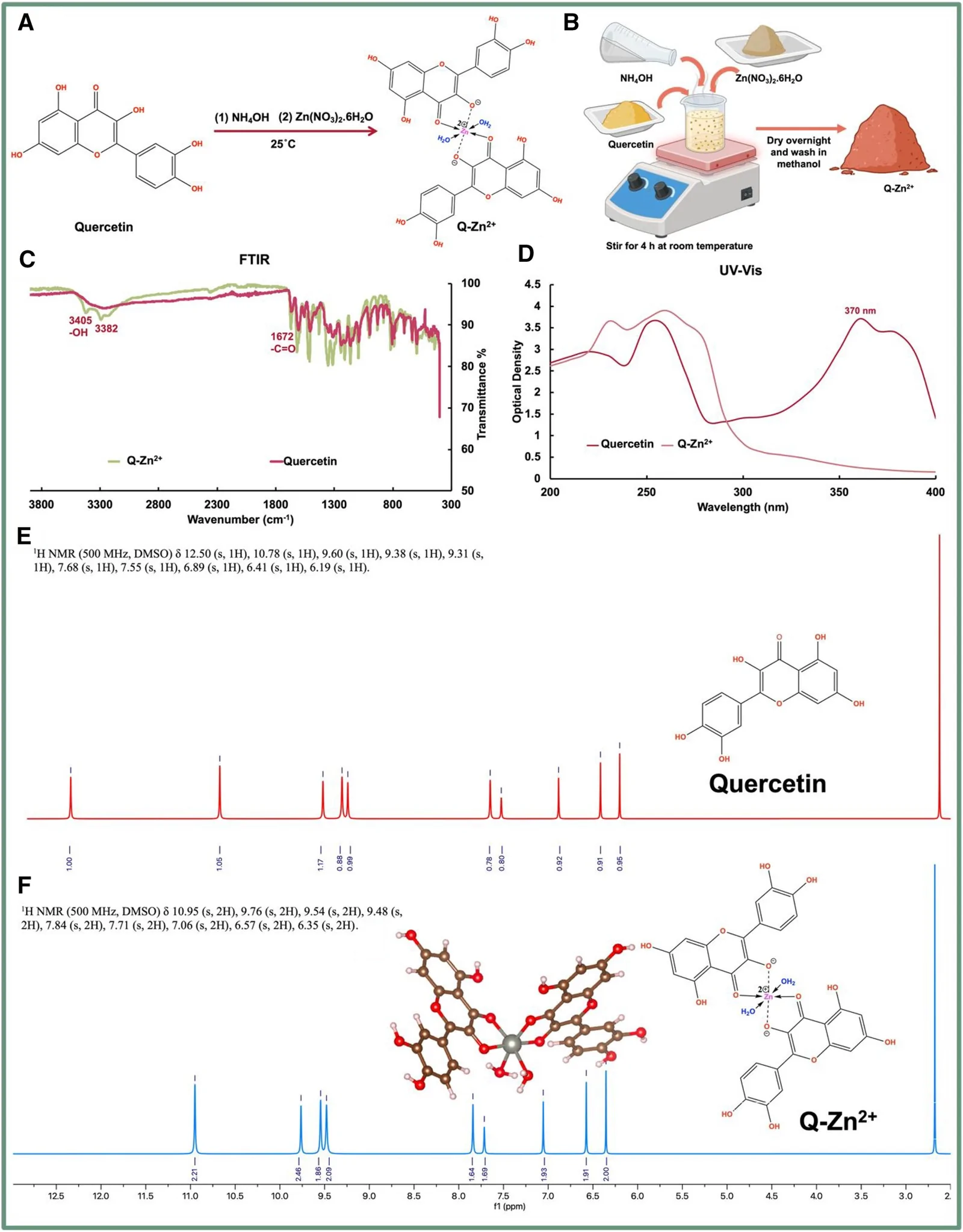
(**A**) Reaction of complex formation. (**B**) Schematic representation of the synthesis. (**C**) FTIR spectra with characteristic peaks of Quercetin and the Q–Zn^2+^. (**D**) UV-Vis spectra of Quercetin and the complex. (**E**) ^1^H NMR of the Quercetin and the complex, done in DMSO. (**F**) The complexation between Zn^2+^ and two molecules of Quercetin using the GFN2-xTB method implemented in the xtb software package.

We studied the complexation between Zn^2+^ and Quercetin using the GFN2-xTB method implemented in the xtb software package. In the initial setup, we included one Zn^2+^ atom, two Quercetin molecules, and two H_2_O molecules, and performed the geometry optimization under neutral conditions with implicit aqueous solvation modeled by the GBSA water approach. The optimized structure of the complex, shown in Figure 1F, indicates that zinc adopts an octahedral coordination geometry by binding four oxygen atoms from the two Quercetin molecules and two oxygen atoms from the water molecules in its primary coordination shell. Our analysis showed that Zn^2+^–O bond lengths are in the range of 1.9–2.2 Å, consistent with expected metal–oxygen distances in such complexes. The Quercetin oxygen atoms are observed closer to Zn (∼2.0 Å) compared to the water oxygen atoms (∼2.25 Å), showing stronger binding between Zn^2+^ and Quercetin compared to water. The analysis of the dihedral angles between the central aromatic planes of the Quercetin ligands (and the corresponding bite angles at Zn^2+^) suggests an orientation of approximately 70–120°, resulting in a nearly perpendicular arrangement (plane–plane dihedral angle ∼90°). This arrangement can be attributed to the steric repulsions of the bulky Quercetin ligands, which in turn control the coordination structure and influence the reactivity and the stability of the overall complex. The thermodynamic stability of the Q–Zn^2+^ complex was evaluated through the ligand-exchange reaction [Zn(H_2_O)_6_]^2+^ + 2Q^−^ → [Zn(Q)_2_(H_2_O)_2_] + 4H_2_O, where Q^−^ represents deprotonated Quercetin. Here, hydrated Zn^2+^ undergoes water exchange during ligand binding, and prior reports show that Quercetin can form 2:1 ligand–metal complexes with Zn^2+^ [44, 45]. The calculated binding free energy of ΔG = −34.4 kcal/mol indicates that the Zn(Q)_2_(H_2_O)_2_ complex is stable in aqueous solution and not prone to spontaneous dissociation under standard conditions.

### Quercetin drug release kinetics

Figure 2A illustrates the proposed mechanism for the release of Quercetin from the complex in the scaffold under different pH conditions. Quercetin contains multiple hydroxyl substituents with distinct pKa values (∼7.1–12.0), whose deprotonation under physiological to alkaline conditions generates phenoxide anions. These deprotonated oxygens are stabilized by intramolecular resonance with the aromatic system [46]. The resulting bidentate coordination forms a stable complex that anchors within the scaffold, limiting premature drug diffusion. Under acidic conditions, protonation of the coordinating phenoxide groups disrupts resonance stabilization and weakens metal-ligand bonding, resulting in dissociation of the complex into free Quercetin and Zn^2+^ ions. Release measurements were Quercetin-related rather than direct measurements of zinc release. The SEM images in Figure 2B were included to illustrate overall surface morphology after release rather than fine microstructural details at high magnification. Compared with the relatively smooth and well-defined scaffold surfaces before release, the samples exposed to pH 5.0 showed increased surface roughness, localized surface erosion, and loss of surface definition, whereas the pH 7.4 samples remained comparatively intact. This suggests that the acidic environment accelerates scaffold degradation, facilitating drug release. The scaffolds maintain structural integrity at physiological pH, indicating a slower breakdown process. Figure 2C and D present the cumulative drug release profiles for Quercetin and the Q–Zn^2+^ complex under pH 5.0 and 7.4 conditions, respectively, over 24 days. The 24-h release profiles at both pHs are shown in Figure 2E and F. The release profile at pH 5.0 shows that Quercetin alone exhibits a rapid initial burst release, reaching approximately 41% over 24 days. This quick release is due to weaker binding interactions within the scaffold and Quercetin’s solubility in the acidic medium. In contrast, the Q–Zn^2+^ complex shows a slower and sustained release of ∼16%. This difference can be attributed to the strong coordination bonds between Quercetin and Zn^2+^, which require greater energy to dissociate, thus controlling the release rate. More images of the scaffold after the release are present in Supplementary Figure S2 at both pH values. The accuracy and precision of the UV-Vis in measuring Quercetin and the Q–Zn^2+^ complex were collected and shown in Supplementary Tables S1 and S2.

**Figure 2:**
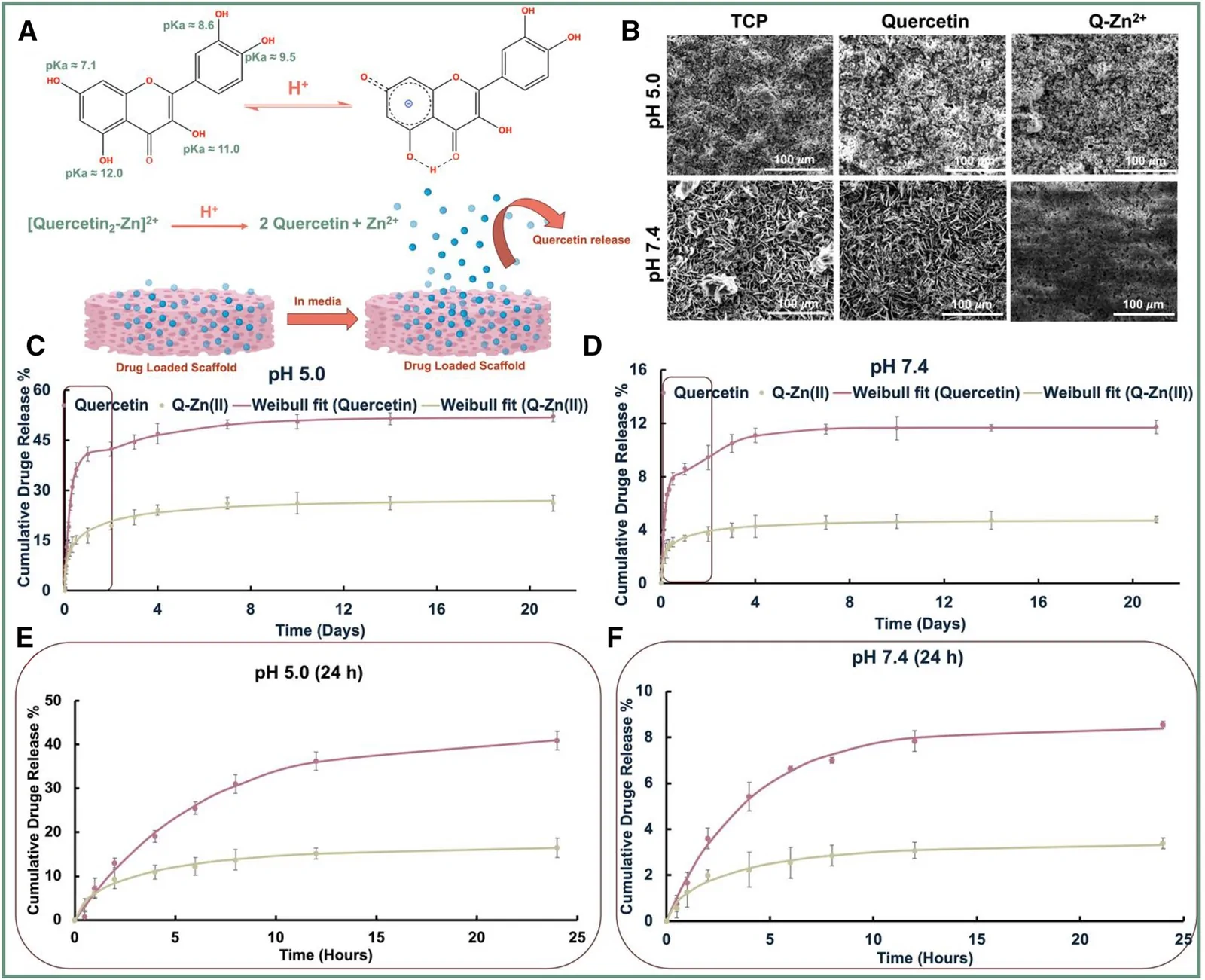
Drug release kinetics study of Quercetin and Q-Zn^2+^ complex at pH 7.4 and pH 5.0. (**A**) Shows the deprotonation of Quercetin in media. (**B**) SEM images of the scaffolds taken after drug release on day 24. (**C**) and (**D**) show the release profile of Quercetin and the complex at pH 5.0 and 7.4 for 24 days, respectively. (**E**) and (**F**) show the release profiles of Quercetin and the complex within 24 h at pH 5.0 and 7.4, respectively. All curve fitting achieved a *R*^2^ value of 0.99 or greater.

At pH 7.4, both Quercetin and the Q–Zn^2+^ complex exhibit reduced release rates. Quercetin exhibits ∼12% release over 24 days, whereas the Q–Zn^2+^ complex displays a more controlled release of ∼5%. This observation supports the hypothesis that the neutral pH stabilizes the complex and prevents rapid dissociation, suggesting its suitability for long-term applications where steady drug release is critical. The release kinetics of Quercetin and the Q–Zn^2+^ complex were analyzed using the Weibull CDF (cumulative distribution function) kinetic model, which is an effective and adequate model for characterizing controlled release mechanisms from ceramic systems. Quercetin’s release profile followed a rapid kinetic pattern, indicative of an initial burst release mechanism due to weaker binding and faster diffusion. However, the Weibull model fitting for the Q–Zn^2+^ complex demonstrated a more gradual, sustained release, confirming a mechanism governed by complex diffusion and delayed dissociation facilitated by the robust coordination between Quercetin and Zn^2+^. Zn^2+^ stabilizes, forming a coordination network that resists rapid breakdown, especially under physiological pH conditions. This slow, controlled release behavior points to the potential of the Q–Zn^2+^ complex for applications requiring sustained drug delivery, particularly in conditions with varying pH levels, such as inflamed surgery sites or tumor microenvironments. The combination of pH-responsive release and prolonged drug availability positions the Q–Zn^2+^ complex as an advantageous system for targeted and efficient therapeutic delivery. The Weibull CDF, F(t), represents the fraction of drug released at time t and follows a stretched exponential behavior (Eq. 2). In this model, the shape parameter (b) describes the drug-release mechanism, while the scale parameter (a) determines the release rate. Values of *b* < 0.75 indicate Fickian diffusion dominated by pore-driven transport, *b* ≈ 1 corresponds to first-order or exponential release, and *b* > 1 suggests a sigmoidal or complex release mechanism, often involving polymer erosion or swelling. The G(t) model describes the total fraction of drug released at time t, characterizing burst or exponential-type release where the release rate is initially high before reaching a plateau (Eq. 3). Here, R denotes the maximum release fraction, c is the scale parameter, and d represents the shape parameter that defines the curve’s steepness.


(2) $$F(t)=1-e^{-{\left(\frac{t}{a}\right)}^{b}}$$

(3) $$G(t)=R(1-e^{{-(\frac{t}{c})}^{d}})$$

At pH 5.0, Quercetin’s best-fit parameters were c=6.1 and d=1.01 in a saturated exponential model for the first 24 h, then a=0.288 and b=3.88 in a Weibull model. Q-${\mathrm{Zn}}^{2+}$ was a=0.704 and b=0.9283. At pH 7.4, Quercetin was best fit using only a Weibull model with a=0.6433 and b=0.5688 for the first 24 h, and a=0.4535 and b=0.5734 for the remainder of the release. Q-${\mathrm{Zn}}^{2+}\;$in pH 7.4 was the best fit with a Weibull CDF using a=0.5351 and b=0.4219.

### *In vitro* OB cell culture study

An 11-day cell culture study evaluated the cytocompatibility of Q–Zn^2+^ loaded 3DP TCP scaffolds using OB cells. The MTT assay results (Figure 3A) showed that no scaffolds exhibited cytotoxic effects. Q–Zn^2+^ scaffolds showed a 42% increase in cell viability compared to TCP, indicating enhanced support for cell proliferation. SEM analysis on day 11 (Figure 3B) showed well-adhered OBs on Q–Zn^2+^ scaffolds, with extended filopodia and flattened morphology. The presence of attached cells was visible across all surfaces, though their density and spreading were most pronounced on Q–Zn^2+^. The live/dead assay results (Figure 3C) support the MTT assay, indicating a high percentage of live cells across all scaffold types. The ALP assay (Figure 3D) showed higher mineralization for both treated scaffolds. On day 11, ALP activity in the Quercetin-loaded and Q–Zn^2+^-loaded 3DP TCP scaffolds was 16% and 29% higher, respectively, compared to TCP scaffolds. Figure 3E quantifies live/dead confocal images, showing that Q–Zn^2+^ scaffolds had a marginally higher percentage of live cells than TCP and Quercetin scaffolds. The improved cell viability in the presence of Q–Zn^2+^ can be attributed to the bioactive properties of zinc, which are known to support osteogenesis and cell proliferation [47].

**Figure 3:**
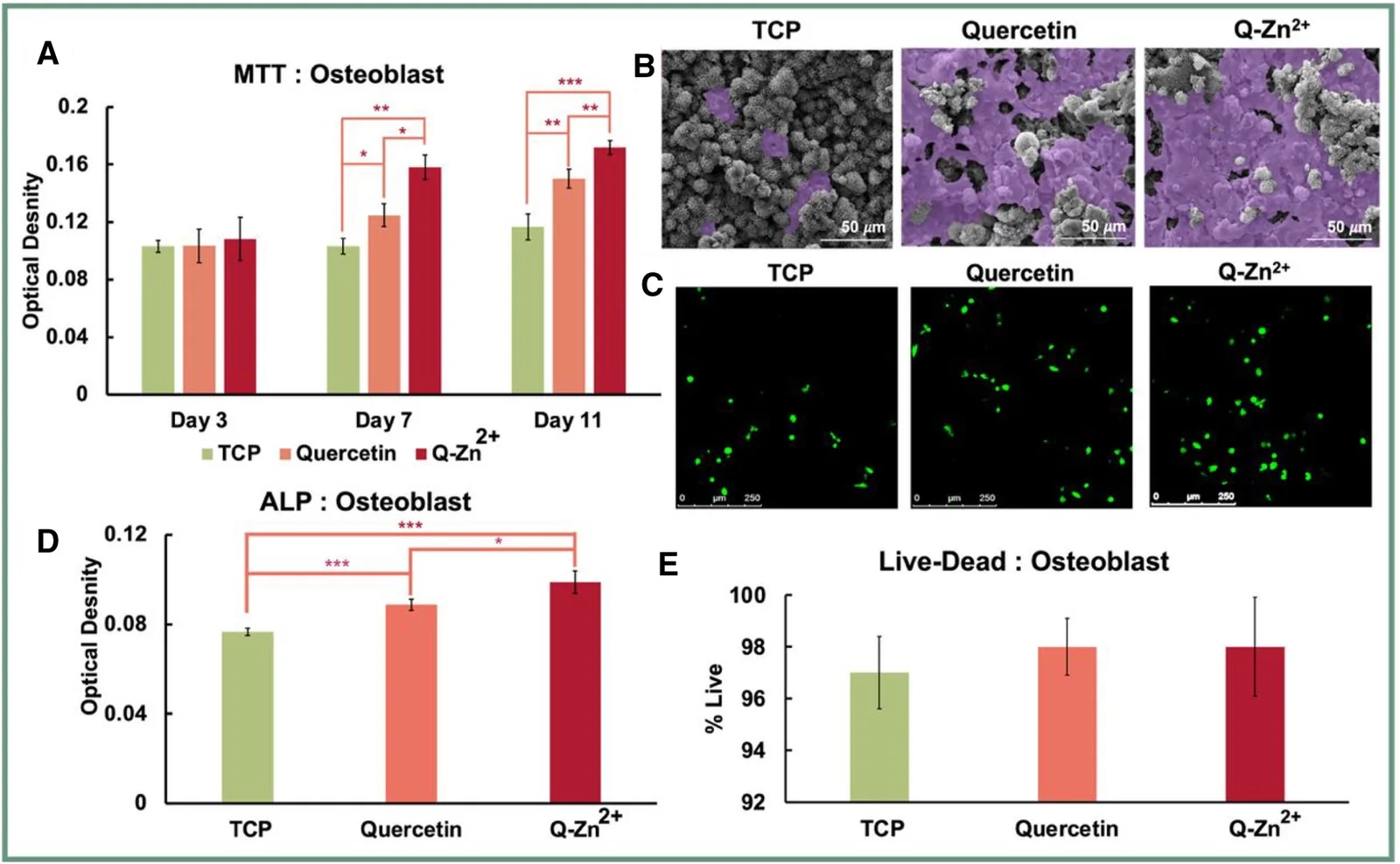
Effect of Q–Zn^2+^ on OB cells. (**A**) MTT assay showing cell viability on days 3, 7, and 11. (**B**) SEM images on day 11 showing cell attachment (cells highlighted in purple). (**C**) Live/dead staining with green indicating viable cells. (**D**) ALP activity on day 11 indicates early osteogenic differentiation. (**E**) Quantification of live cell percentage (*P < 0.05, **P < 0.01, ***P < 0.001, *n* = 3).

### *In vitro* OS cell culture

The MTT assay results presented in Figure 4A assess OS cell viability on TCP, Quercetin, and Q–Zn^2+^ scaffolds over 3, 7, and 11 days. Initial observations on day 3 show no substantial difference in cell viability among the treatments. By day 7 and continuing through day 11, Quercetin and Q–Zn^2+^ scaffolds demonstrated a considerable decrease (∼55%) in cell viability compared to TCP. This suggests that the Quercetin and Q–Zn^2+^ complexes exert cytotoxic effects on OS cells over time, potentially inhibiting their proliferation and metabolic activity. JC-1 membrane staining was taken on day 11 to evaluate the potential gradient across the OS cell membrane and is provided in Figure 4B. The TCP sample was observed to have greater red fluorescence due to the accumulation of the monomer, which formed J-aggregates in healthy OS membranes. Green fluorescence was observed in the treatment samples, which is caused by a low membrane potential, indicating that unhealthy cells are undergoing cell apoptosis. Live/dead assay results, shown in Figure 4C, confirm cytotoxicity. Red fluorescence represents dead cells, indicating that Quercetin and Q–Zn^2+^ scaffolds induce higher levels of cell death than TCP. Quantitative analysis of the live/dead assay (Figure 4D) indicated a reduction in the percentage of live cells for Quercetin and Q–Zn^2+^. SEM images (Figure 4E) show the surface morphology of the OS cells on TCP and Quercetin, and Q–Zn^2+^-loaded scaffolds. On TCP, OS cells appeared healthy, rounded, and well-attached. In contrast, cells on Quercetin and Q–Zn^2+^ scaffolds appeared unhealthy, with a ruptured morphology (as highlighted in red) and loss of cellular integrity, consistent with apoptosis-induced morphological degradation. No aggregation or healthy attachment was seen in treated groups, reinforcing their cytotoxic efficacy.

**Figure 4:**
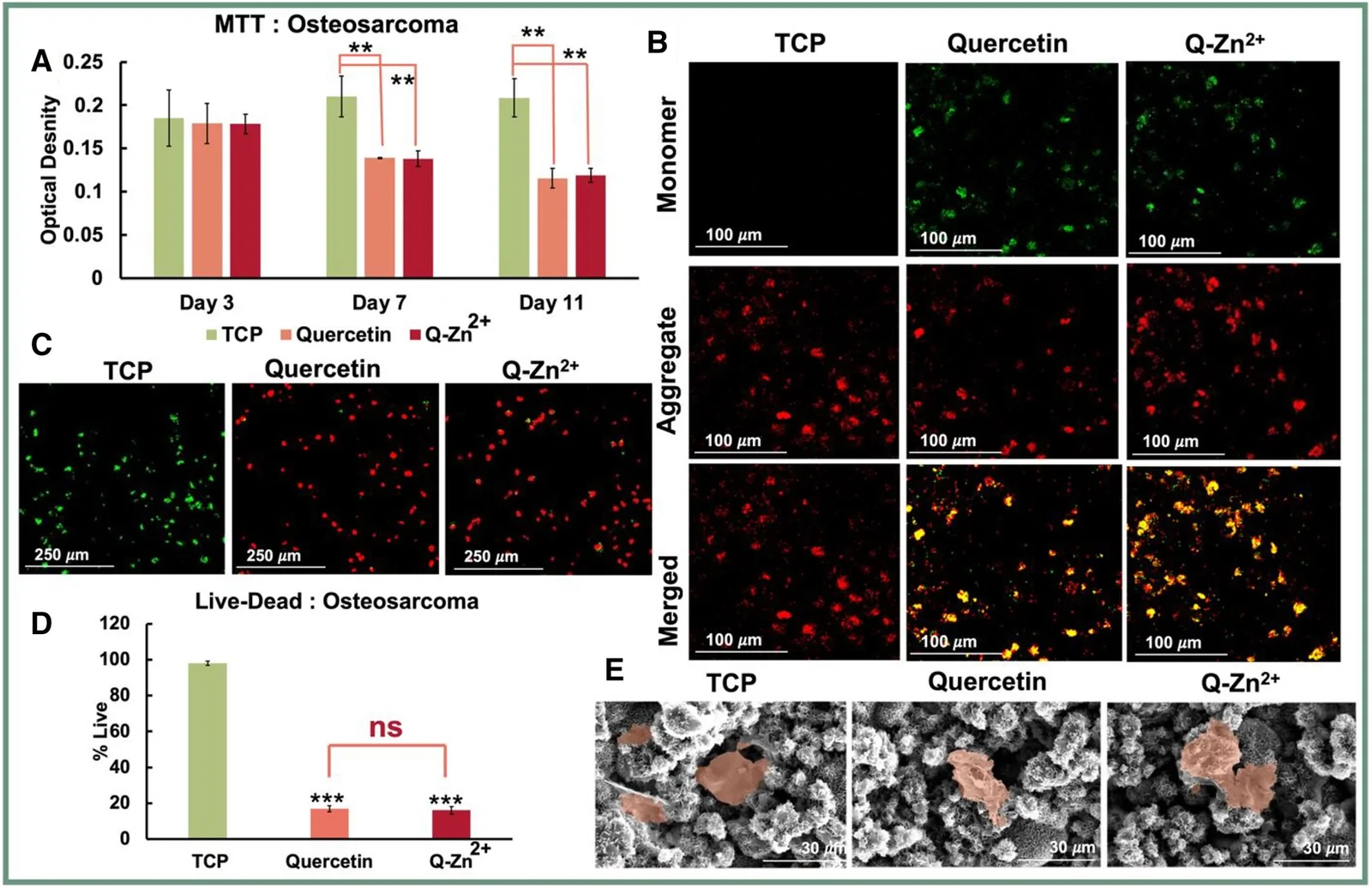
Evaluation of OS cell response to Quercetin and Q–Zn^2+^-loaded scaffolds. (**A**) MTT assay showing cell viability at days 3, 7, and 11. (**B**) JC-1 staining on day 11 indicates mitochondrial membrane potential changes (red = healthy; green = unhealthy). (**C**) Live/dead staining shows increased cytotoxicity in treated groups. (**D**) Quantification of live cell percentage with the p-star values indicating comparison to TCP and ns = not significant. *** indicates a p-value < 0.001. (**E**) SEM images showing cell morphology on scaffold surfaces (cell highlighted in red) (**P < 0.01, ***P < 0.001 vs. TCP, *n* = 3).

### *In vitro* antibacterial efficacy of Q–Zn^2+^ complex

The antibacterial effects of Quercetin and Q–Zn^2+^ from TCP scaffolds were evaluated against *S. aureus* at 36 and 72 h. Figure 5A presents a schematic showing bacterial inhibition by Q–Zn^2+^ scaffolds. The agar plates (Figure 5B) show a visible difference in bacterial colony formation across treatments. Both Quercetin and Q–Zn^2+^ loaded scaffolds showed reductions in bacterial growth compared to TCP, at both time points. The antibacterial efficacy (Figure 5C) was calculated based on agar plate images at both time points. The Q–Zn^2+^ showed the highest antibacterial efficacy (∼93%), which was found to be higher than Quercetin (∼80%). This suggests that Zn^2+^ might also play some role in enhancing the antibacterial properties through synergistic effects. SEM images (Figure 5D) provide insights into the bacterial interaction with scaffold surfaces. At 36 h, Quercetin and Q–Zn^2+^ scaffolds displayed disrupted bacterial cell membranes compared to the intact bacteria on the TCP surface. By 72 h, the Q–Zn^2+^ scaffolds showed more dead bacterial cell debris, indicative of extensive cell lysis. The unhealthy bacteria are highlighted in green, while the healthy bacteria are highlighted in blue. This effect was less pronounced on the Quercetin, reinforcing the superior antibacterial activity of the Q–Zn^2+^ complex. Live/dead images in Figure 5E show the health status of bacteria on the scaffolds, with green fluorescence indicating live cells and red fluorescence indicating dead cells. Q–Zn^2+^ loaded scaffolds showed higher red fluorescence at both time points, indicating extensive bacterial death. Quantification in Figure 5F confirms these observations, with the percentage of live bacteria dropping for both Quercetin and Q–Zn^2+^ scaffolds compared to TCP.

**Figure 5:**
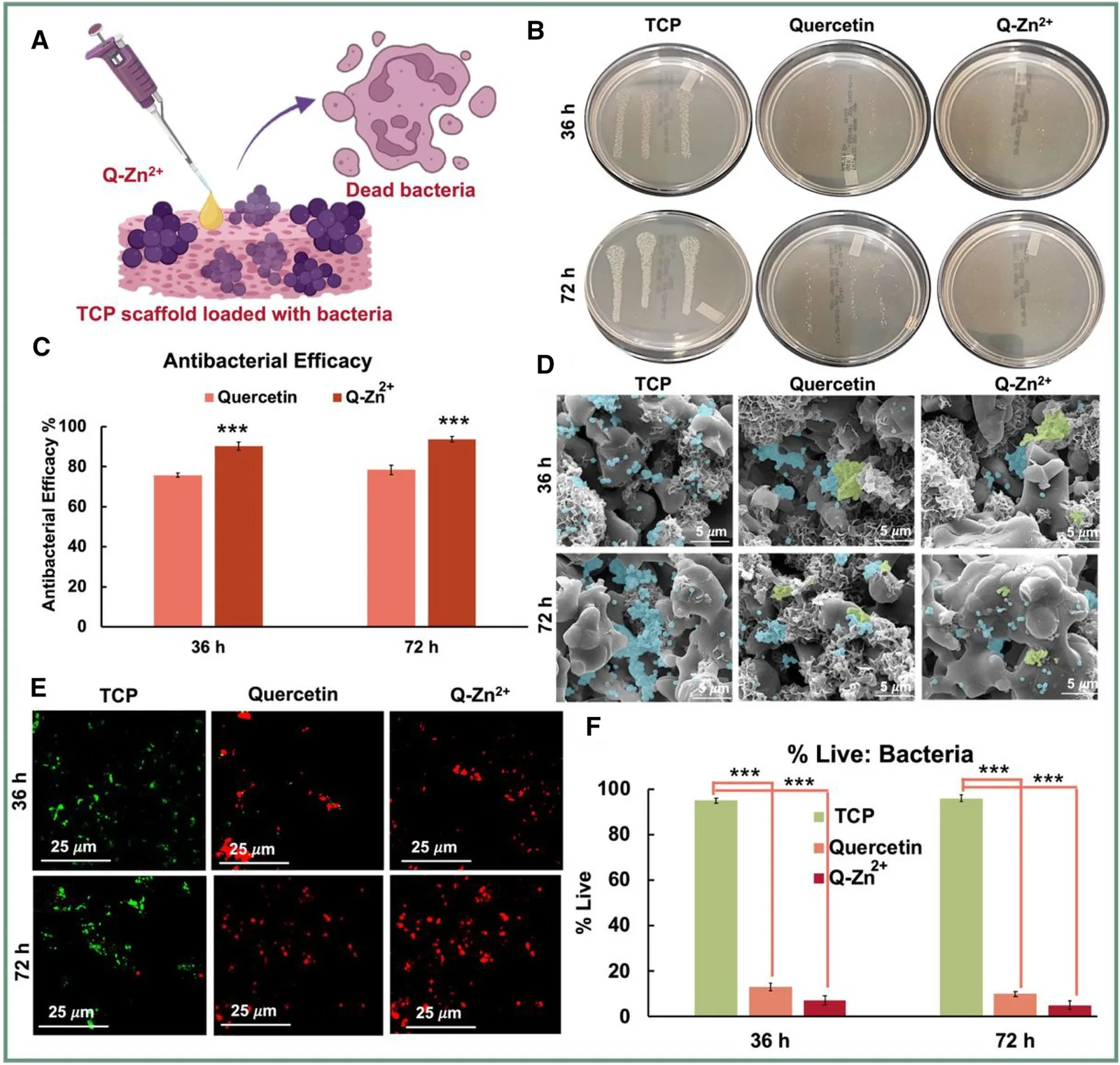
Antibacterial evaluation of Quercetin and Q–Zn^2+^-loaded 3DP TCP scaffolds against *S. aureus*. (**A**) Schematic of proposed antibacterial inhibition. (**B**) Agar plate images at 36 and 72 h. (**C**) Quantification of agar plates displaying CFU-based antibacterial efficacy. (**D**) SEM images showing bacterial morphology (healthy bacteria in blue and unhealthy cells in green). (**E**) Live/dead staining of bacterial cells. (**F**) Quantification of live/dead fluorescence signals (***P < 0.001 vs. TCP control, *n* = 3).

## Discussion

Developing bioactive scaffolds coupled with controlled drug release remains a primary clinical challenge. Over 7 000 000 cases of CMF disorders are reported annually and are expected to grow due to an aging population, which intensifies the demand for scaffolds with advanced drug delivery capabilities [1]. Quercetin contains multiple hydroxyl and carbonyl groups capable of chelating metal ions, making it suitable for forming stable complexes [48]. It was complexed with Zn^2+^ to enhance its chemical stability, enable sustained release, and synergistically promote osteogenesis while maintaining selective cytotoxicity toward OS cells and bacterial cells. ^1^H NMR spectra showed that hydroxyl groups form coordinated bonds with the Zn^2+^. FTIR spectroscopy confirmed complexation, with shifts in the O–H (3405 and 3382 cm^−1^) and C=O (1672 cm^−1^) stretching bands indicating coordination between Quercetin and Zn^2+^. The coordination of Quercetin to Zn^2+^ displaces nitrate ions, increasing system entropy and reducing Gibbs free energy, thereby contributing to the thermodynamic stability of the complex. The formation of the Q–Zn^2+^ complex was supported by FTIR, NMR, and UV-Vis characterization, as well as the formation of an insoluble precipitate during synthesis. The GFN2-xTB model was therefore used as a representative proposed structure for a 1:2 Zn:Quercetin coordination environment with water coordination. However, this model should not be interpreted as definitive confirmation of exclusive product stoichiometry or complete chemical purity. Additional characterization by mass spectrometry and/or elemental analysis would be useful in future work to further confirm molecular composition and detect potential minor residual species.

The Q–Zn^2+^ complex exhibited sustained and pH-responsive release, indicating improved chemical stability compared to free Quercetin. This is due to the strong coordination between Zn^2+^ and Quercetin, which reduces the rate of dissociation and delays the diffusion of the drug. In contrast, free Quercetin showed a burst release due to weak adsorption with TCP. Deprotonation of Quercetin hydroxyl groups with pKa 7.1 generates resonance-stabilized phenoxide anions that act as strong electron donors to Zn^2+^, forming a stable bidentate complex and retaining the drug within the scaffold [46]. Acidic protonation disrupts this resonance stabilization, weakens Zn^2+^ coordination, and drives dissociation into free Quercetin and Zn^2+^ ions, enabling controlled release. Under acidic conditions, protonation of coordinating groups weakens the metal–ligand bond, resulting in faster release. At physiological pH, the complex remains intact, allowing gradual and prolonged drug release. The Weibull CDF model fitting and the saturated exponential indicated Fickian diffusion (*b* < 1, *d* < 1) for both the complex and Quercetin at pH 7.4, suggesting release is primarily controlled by diffusion through the scaffold. The TCP scaffold’s microporous structure supports this mechanism by enabling slow, continuous drug transport. Together, complexation and scaffold architecture suppress initial burst release and provide a controlled, localized delivery profile. This combination of pH sensitivity and sustained release is instrumental in bone repair, where local acidosis occurs in inflamed or tumor-associated tissue. The Q–Zn^2+^ system enables targeted drug delivery in such environments, reducing off-target exposure and supporting long-term therapeutic effects. Although Q–Zn^2+^ complex formation was supported by spectroscopic characterization, Zn^2+^ release and residual post-release Zn content were not directly quantified in the present study. Therefore, the release results should be interpreted primarily as Quercetin-associated release behavior. Future studies should include elemental analysis of release media and digested post-release scaffolds, such as ICP-OES or ICP-MS, to determine the coupled release and retention profiles of Quercetin and Zn^2+^.

To assess the osteogenic viability of the Q–Zn^2+^ complex, an 11-day culture was conducted using the OB cell line. A cytotoxicity threshold experiment using the MTT assay was conducted to evaluate the effective dose of the complex loaded on TCP (Supplementary Figure S1). The results showed 10 µg to have the best OB response, which is in line with previously reported work that supports the biological relevance of low-dose Q–Zn exposure, while our study identified the optimal loading for TCP scaffolds [25]. Metabolic activity, assessed via the MTT assay on days 3, 7, and 11, showed a sustained increase, with Q–Zn^2+^ -treated scaffolds consistently supporting higher viability than both TCP and Quercetin. Live/dead staining at day 7 showed a higher density of viable cells on Q–Zn^2+^ scaffolds. Similarly, SEM images at day 11 showed well-adhered OBs with extended filopodia, indicating healthy attachment and a favorable scaffold interaction. ALP activity, measured on day 11, was also higher in the Q–Zn^2+^, consistent with early-stage osteogenic differentiation. Higher ALP activity in the treated groups indicates enhanced early OBs maturation [49]. This response likely stems from the synergistic effects of Zn^2+^, which acts as a cofactor in bone-related enzymatic pathways, and Quercetin, which supports cellular redox balance [18, 9, 50]. ALP is an enzyme crucial for hydrolyzing phosphate esters in organic molecules, thereby facilitating the deposition of calcium phosphate, which is essential for bone matrix formation [51–53].

The anticancer effects of the Q–Zn^2+^ complex were studied over 11 days using OS cell cultures. On day 3, cell viability remained comparable across all groups, suggesting that the complex does not induce immediate cytotoxic effects. By day 7, a reduction in viability was observed in the Q–Zn^2+^-loaded samples, which became more pronounced by day 11, resulting in ∼55% reduction compared to the TCP. This gradual reduction indicates a delayed but sustained inhibitory effect on OS proliferation. Live/dead staining at day 11 confirmed this trend, with visibly more red-stained cells on Q–Zn^2+^ scaffolds. JC-1 staining showed increased green fluorescence in treated samples, reflecting mitochondrial membrane disruption, a key indicator of apoptosis. These observations align with Quercetin’s known ability to modulate intracellular oxidative stress and trigger intrinsic apoptotic pathways. Previous studies suggest Quercetin induces apoptosis via the Bax/Bcl-2 axis and mitochondrial disruption. The observed reduction in OS viability is likely aligned with this mechanism [54, 55]. This behavior contrasts with the response of OBs cultured under the same conditions. Cancerous cells like OS have higher reactive oxygen species (ROS) levels due to their high proliferation rate [56]. During the process of an antioxidant reducing ROS intracellularly, the species are broken into O_2_ and H_2_O_2_, which temporarily increases ROS [57]. In healthy cells like OBs, this temporary increase is fine; however, in OS, the increase pushes the cell past the safe threshold [56]. This results in oxidative stress damaging the OS cells. While OS viability decreased over time, OB cell viability and differentiation markers, including ALP, progressively increased on days 3, 7, and 11. The ability of Q–Zn^2+^ to suppress tumor cells while supporting healthy bone cell function highlights its potential as a selective and localized treatment strategy for bone repair in cancer-associated defects.

The Q–Zn^2+^ complex showed antibacterial activity against *S. aureus.* Zn^2+^ plays a dual role at low concentrations, promoting cellular function, while at higher levels (above 0.01–0.1 mM), it exerts bacteriostatic and bactericidal effects [58, 59]. The positive charge on the Zn^2+^ allows it to bind to the negatively charged surface of bacteria through the teichoic acid in the peptidoglycan layer and the lipoteichoic acid on the cell membrane [60]. The subsequent electrostatic interactions lead to a potential gradient across the bacterial membrane, resulting in damage to the surface [61, 62]. SEM images of Q–Zn^2+^ samples show ruptured bacterial morphology compared to Quercetin-treated samples, indicating Zn^2+^ involvement in membrane disruption. The passive diffusion of Zn^2+^ into bacteria results in structural damage [63]. Trace metals, such as Zn^2+^, play a crucial role in maintaining healthy cellular function. Still, at elevated levels, they can displace ligands that typically bind to other trace metals, leading to an overproduction of ROS [64]. This ROS overproduction damages DNA and mRNA functions [65]. Quercetin interacts with bacterial cell membranes of *S. aureus* [66]. This interaction causes structural changes and destabilizes the cell wall, leading to the leakage of intracellular components and ultimately resulting in bacterial death [67, 68]. Live/dead staining confirmed bacterial membrane rupture and death on Q–Zn^2+^ scaffolds, consistent with the synergistic antibacterial activity of Quercetin and zinc. The Q–Zn^2+^ complex supports bone regeneration, inhibits tumors, and reduces bacterial load, making it a strong candidate for orthopedic implants in cancer-associated bone defects.

## Limitations and future work

This study demonstrates the *in vitro* release behavior and biological activity of Q–Zn^2+^-loaded 3DP TCP scaffolds; however, several limitations should be considered. First, the release experiments were performed under simplified *in vitro* conditions and do not fully capture the dynamic fluid exchange, protein adsorption, inflammatory environment, cellular uptake, degradation, or clearance mechanisms present *in vivo*. Therefore, although cumulative release from the scaffolds remained limited over 24 days under the present conditions, these data should not be interpreted as direct evidence of *in vivo* safety or controlled systemic exposure. In particular, systemic Zn^2+^ toxicity risk, biodistribution, local tissue accumulation, immune response, and organ-level clearance were not evaluated in this study. Future work should include *in vivo* implantation studies to assess local bone regeneration, anti-OS response, systemic Zn^2+^ concentrations, organ accumulation, inflammatory response, and long-term biosafety. Zn^2+^ release should be directly measured using ICP-OES or ICP-MS as it is released from the scaffold.

Hemocompatibility and immunogenicity were beyond the scope of the present study and were not directly assessed. While the scaffold is intended for local bone-defect implantation rather than direct blood-contacting applications, blood compatibility and immune response remain important considerations for future translation. Therefore, future studies should include hemolysis assays and cytokine profiling in relevant immune-cell models to evaluate potential blood compatibility concerns and inflammatory responses associated with Q–Zn^2+^-loaded TCP scaffolds.

## Conclusion

This work leveraged metal-ligand chelation in a 3DP TCP scaffold using a Q–Zn^2+^ complex to achieve controlled therapeutic delivery and multifunctional osteogenic performance. Q–Zn^2+^ chelation significantly reduced bacterial growth and OS cell viability while enhancing cytocompatibility toward OBs when incorporated into 3DP TCP scaffolds. Release kinetics demonstrated a controlled and sustained release over 24 days, minimizing burst release and improving therapeutic effectiveness. The Q–Zn^2+^ complex promoted higher OB viability, proliferation, and mineralization compared to controls. In OS studies, the complex induced a 55% reduction in cell viability by day 11, accompanied by increased membrane permeability and apoptosis, as confirmed by live/dead staining and JC-1 assays. Strong antibacterial activity was also observed, with a 93% reduction in bacterial viability within 72 h. Collectively, these results highlight the multifunctional potential of the Q–Zn^2+^ complex for bone regeneration, antibacterial scaffolds, and anticancer applications. This work supports the broader utility of chelated natural metal complexes as effective therapeutic strategies for enhancing bone tissue repair.

## Supplementary Material

rbag139_Supplementary_Data

## Data Availability

The supporting data for this study are available from the corresponding authors upon reasonable request.
