# Supplementary material for: 3D-printed tricalcium phosphate scaffolds with Quercetin-zinc chelation for osteogenic, antibacterial and anti-osteosarcoma activity
Source: Regen Biomater. 2026 Oct 16;13:rbag139. doi: 10.1093/rb/rbag139 (PMC13677079; doi:10.1093/rb/rbag139)
Supplement: Supplementary file 1 [file rbag139_supplementary_data.docx]

**3D Printed Calcium Phosphate Scaffolds with Quercetin-Zinc Chelation for Osteogenic and Antibacterial Activity**

**Susmita Bose* ^1^, Connor Toulou ^1^, Aditi Dahiya ^1^**

^1^ W. M. Keck Biomedical Materials Research Laboratory, School of Mechanical and Materials Engineering, Washington State University, Pullman, WA 99164, USA. [sbose@wsu.edu](mailto:sbose@wsu.edu)*, [connor.toulou@wsu.edu](mailto:connor.toulou@wsu.edu), [aditi.dahiya@wsu.edu](mailto:aditi.dahiya@wsu.edu).

**Supplementary Information**

**Figure S1** - Effective concentration study results showing the effect of the Q-$\mathrm{Zn}^{2+}$complex on OB cells at varying concentrations. 10 $\mu$g was determined to perform the best and was thus selected. All concentrations were loaded on 3DP TCP. (*p < 0.05, *p < 0.01, ***p < 0.001, n=3).

**Figure S2** - Optical images of samples before (TCP) and after release in pH 5.0 and 7.4. Erosion marks can be seen on the Quercetin samples and in select locations on the pH 5.0 Q-Zn^2+^ sample.

**Table S1** – Accuracy and intraday precision measurements.

$$\% Accuracy=\frac{Measured Concentration}{Theoretical Concentration}\times100$$

$$\% Relative Standard Deviation \left( RSD \right)=\frac{Standard Deviation}{Average}\times100$$

**Table S2** – Interday precision measurement.
